# Prenatal Risk Factors for Adverse Developmental Outcome in Preterm Infants—Systematic Review

**DOI:** 10.3389/fpsyg.2019.00595

**Published:** 2019-03-26

**Authors:** Milla K. Ylijoki, Eeva Ekholm, Mikael Ekblad, Liisa Lehtonen

**Affiliations:** ^1^Department of Paediatrics and Adolescent Medicine, Turku University Hospital, University of Turku, Turku, Finland; ^2^Department of Paediatric Neurology, Turku University Hospital, University of Turku, Turku, Finland; ^3^Department of Obstetrics and Gynaecology, Turku University Hospital, University of Turku, Turku, Finland; ^4^Department of Human Development and Family Studies, Purdue University, West Lafayette, IN, United States; ^5^Department of General Practice, Turku University Hospital, Turku University, Turku, Finland

**Keywords:** chorioamnionitis, smoking, doppler, preterm, development

## Abstract

**Background:** Preterm infants are still at an increased risk for suboptimal neurodevelopmental outcomes when compared with term born infants. The development of a child born preterm can be jeopardized by suboptimal conditions during pregnancy, in addition to the suboptimal growth environment postnatally compared to the normal *in utero* environment. This review summarizes the literature on the role of chorioamnionitis, placental insufficiency, and maternal smoking on the developmental outcomes of preterm infants.

**Methods:** A systematic database search was performed to identify all original articles published on or before September 12, 2018 that evaluated the impact of clinical or histological chorioamnionitis, abnormal prenatal fetal and placental blood flow, and prenatal smoking exposure on the neuropsychological and cognitive outcomes of preterm infants. We identified a total of 54 studies. Thirty five original articles evaluated the effects of clinical or histological chorioamnionitis; 15 studies evaluated the effects of abnormal blood flow patterns; and four studies evaluated the effects of maternal smoking during pregnancy.

**Results:** The studies on prenatal risk factors showed conflicting results about the impact on the neurodevelopment of preterm infants. The majority of the studies did not show that chorioamnionitis poses a direct risk to the development of preterm infants. The role of abnormal prenatal placental and fetal blood flow on the development of preterm infants remained inconclusive because the sample sizes were often small and methodological problems complicated the interpretation of the data. Maternal smoking during pregnancy was assessed only in one cohort which showed that maternal smoking is a risk for suboptimal cognitive and neuropsychological development in preterm infants.

**Conclusions:** This review summarizes the data on several prenatal risk factors which play a role in the developmental outcomes of preterm infants. To optimize the developmental outcomes, we need to first optimize the fetal wellbeing before birth. More research that extends from the fetal life to long-term developmental outcomes is needed.

## Introduction

Although the perinatal and neonatal care of preterm infants is constantly improving, preterm infants are still at increased risk for suboptimal cognitive and neuropsychological outcomes when compared with term infants. Preterm infants will inevitably have a different developmental environment compared to their physiological *in utero* environment, which poses a risk for the developing brain. This risk increases further with decreasing gestational age (Munck et al., 2010; Lind et al., 2011; Cheong et al., 2017; Hirvonen et al., 2017; Luu et al., 2017; Twilhaar et al., 2018). In addition to medical risk factors associated with preterm birth and intensive care needed in these situations, the premature babies are exposed to other risk factors associated with poor neurodevelopmental outcome, such as parental separation, stress, anxiety, and depression. Also the ability to succesfully breast feed the baby is often compromised in a case on very preterm delivery, which is an additional risk factor for mother-infant interaction and neurodevelopment (Flacking et al., 2012). Further more, there are several prenatal factors related to prematurity which have been suggested to increase the risk of developmental deficits in preterm infants. This review summarizes the research of prevalent prematurity associated prenatal risk factors with accumulating new research on later cognitive and neuropsychological outcomes of preterm infants.

Choriamnionitis is an important cause of preterm delivery. The incidence of chorioamnionitis increases with decreasing gestational age, with nearly all spontaneous preterm deliveries occurring around 24 weeks of gestation being associated with chorioamnionitis (Andrews et al., 2000; Goldenberg et al., 2000, 2008; Goldenberg, 2002). Although some studies have shown that histological and/or clinical chorioamnionitis are associated with suboptimal neurodevelopment in preterm infants, the findings are inconsistent as shown in previous reviews (Ylijoki et al., 2012; van Vliet et al., 2013; Maisonneuve et al., 2017). Whether chorioamnionitis leads to impaired outcomes compared to non-exposed preterm infants born at similar gestational age remains unclear. As inflammation may enhance maturation, chorioamnionitis may also have beneficial effects for preterm infants.

An abnormal placental and fetal blood flow pattern has been shown to occur in about 20% of all very preterm births (Leppänen et al., 2009). It is well known that increased impedance in the umbilical artery flow is associated with an increased perinatal mortality and morbidity, especially in growth restricted fetuses (Karsdorp et al., 1994). To cope with placental insufficiency, the fetus increases blood flow to the brain. This so called “brain sparing” is reflected as an increased ratio between umbilical artery (UA) and middle cerebral artery (MCA) pulsatile indices. Brain sparing has been associated with decreased total brain volume, cortical gray matter volume, and cerebral volumes (Tolsa et al., 2004; Maunu et al., 2007), as well as increased incidence of brain pathology (Leppänen et al., 2009). It has been hypothesized that impaired fetal brain growth may lead to an impaired neurocognitive outcome. However, the data are inconclusive, and there are no previous reviews highlighting the matter.

Maternal smoking during pregnancy is the most common preventable factor causing adverse effects on fetal development. It is associated with an increased risk of preterm birth and low birth weight (Andres and Day, 2000). The risk of sudden infant death syndrome has also been associated with maternal smoking during pregnancy (Mitchell and Milerad, 2006). In addition, maternal smoking has been associated with adverse effects on fetal brain development in term and preterm infants (Ekblad et al., 2010, 2015), and with long term adverse effects in exposed infants, such as psychiatric morbidity (Ekblad et al., 2010) and neurodevelopmental problems (Clifford et al., 2012; Polanska et al., 2015). Study populations have mainly been full-term children. In preterm children, the association of maternal smoking during pregnancy with cognitive and neuropsychological outcomes is less studied, and there are no previous reviews concerning the matter.

In this review, we aim to evaluate the significance of selected prenatal risk factors related to prematurity, such as smoking during pregnancy, abnormal prenatal blood flow patterns, and chorioamnionitis, on the neurodevelopment of preterm children. This information is important for the professionals involved in the follow-up of pregnant women, as well as the professionals, such as psychologists, speech therapists, pediatricians, child neurologists and teachers who are involved in the follow up, therapy and education of preterm children.

## Methods

We performed a systematic electronic database search in the PubMed database (including MeSH search) to identify all original articles published on or before September 12, 2018 that evaluated the impact of clinical or histological chorioamnionitis, prenatal smoking exposure, and abnormal prenatal fetal and placental blood flow on neuropsychological and cognitive outcomes in preterm infants. With chorioamnionitis, the search terms used for the search were *Chorioamnionitis* combined with *Development*. Articles published before October 5, 2011 were part of a previously published review article (Ylijoki et al., 2012). We chose to use the same search terms to be able to combine the search results and update the previous findings with recent publications. With prenatal blood flow (Doppler velocimetry), the search terms used were *doppler* combined with *cognitive outcome* and *placental doppler* combined with *outcome*. With maternal smoking the search terms used for the search were *Smoking, Pregnancy* and *Neurodevelopment* or *Cognitive Development*. In addition, we performed a manual search of the reference lists of all included articles from the database search.

### Study Selection

**Figure 1:**
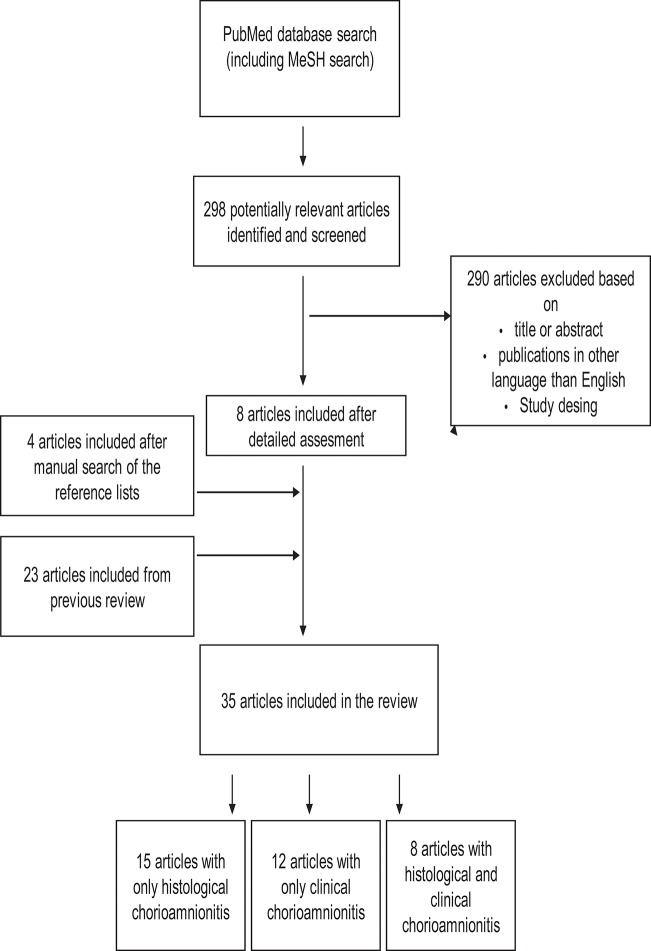
Study selection process for articles about the association between chorioamnionitis and cognitive and neuropsychological outcome in preterm infants. The search was performed for articles published between October 5, 2011and September 12, 2018. Articles published before October 5, 2011 was identified earlier as a part of our previous review.

**Figure 2:**
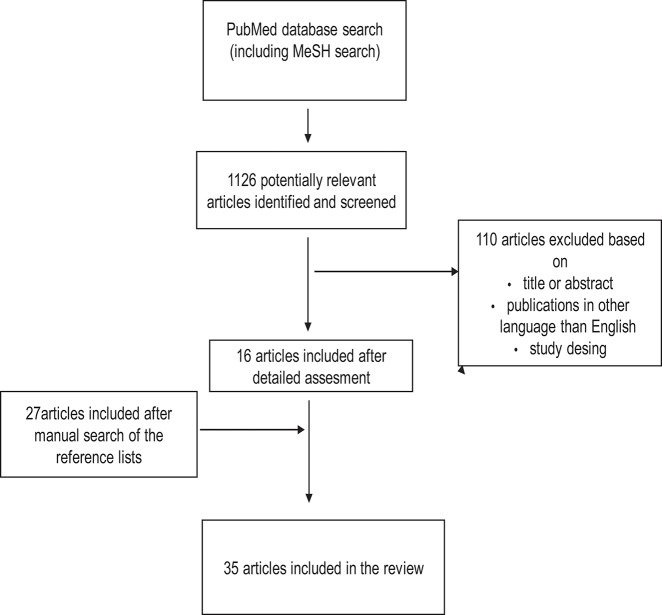
Study selection process for articles about the association between abnormal placental and fetal blood flow and cognitive and neuropsychological outcome in preterm infants.

**Figure 3:**
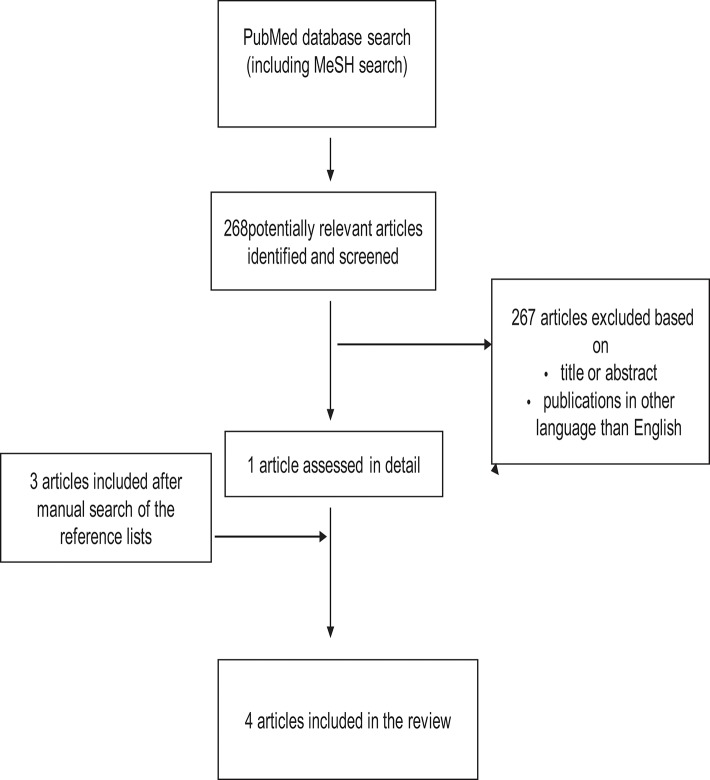
Study selection process for articles about the association between smoking during pregnancy and cognitive and neuropsychological outcome in preterm infants.

In the first phase, the publications were selected based on titles and abstracts to exclude irrelevant publications. Only publications written in English were included. Based on the full text articles, publication were excluded if they did not provide answer to the question of interest. Figures 1–3 show the flow chart of the literature search.

We only included articles with preterm infants (born before 37 weeks of gestation). The developmental outcomes in the included articles were neuropsychological and cognitive development. The methods of evaluation varied greatly among the different studies.

Chorioamnionitis (i.e., inflammation of the fetal membranes) can be classified as either histological or clinical chorioamnionitis. Clinical chorioamniotis is usually diagnosed based on a combination of clinical signs (ruptured membranes, maternal or fetal tachycardia, maternal leukocytosis, foul-smelling amnionitic fluid), but the criteria of the diagnosis in the articles was variable. Histological chorioamnionitis is based on the appearance of neutrophils in the placental tissue. Histological chorioamnionitis can be further classified as maternal or fetal chorioamnionitis (also called funisitis).

Studies using doppler ultrasound to assess fetoplacental blood flow from the umbilical artery, fetal median cerebral artery, and/or the aortic isthmus were included in this review.

All of the articles about the effects of smoking were about maternal smoking during pregnancy.

## Results

We identified 54 studies about prenatal risk factors related to prematurity and their impact on cognitive and neuropsychological outcome. Thirty-five original articles evaluated the effects of clinical or histological chorioamnionitis; 15 studies evaluated the effects of abnormal prenatal Doppler velocimetry; and four studies evaluated the effects of maternal smoking during pregnancy.

### Chorioamnionitis

The majority of the studies found no independent effect of chorioamnionitis on abnormal neurodevelopment (Morales, 1987; Dexter et al., 1999, 2000; Ambalavanan et al., 2000; Kosuge et al., 2000; Vermeulen et al., 2001; Dammann et al., 2003; Kent et al., 2005; Polam et al., 2005; Mu et al., 2007; Redline et al., 2007; Andrews et al., 2008; Helderman et al., 2012; Nasef et al., 2013; Soraisham et al., 2013; Manuck et al., 2014; Pappas et al., 2014; Källén et al., 2015; Miyazaki et al., 2016; Vander Haar and Gyamfi-Bannerman, 2016; Bierstone et al., 2018). There was a similar proportion of studies without association in the groups of histological and clinical chorioamnionitis. However, all studies with associations showed that chorioamninitis was a risk factor, not a protective factor, for later development. The results of the studies about chorioamnionitis are summarized in Table 1.

**Table 1:** The outcomes of the 35 included articles divided according to the definition of chorioamnionitis (clinical, histological or clinical, and/or histological) and the association with developmental outcomes (abnormal cognitive outcome ≤/>2 years of age or other).

			**Abnormal cognitive outcome ≤2 years of age**	**Abnormal cognitive outcome >2 years of age**	**Other**
			**OR (95% CI)**	**OR (95% CI)**	**OR (95% CI)**
Histological chorioamnionitis *n* = 20	VLBW/VLGA infants *n* = 18	(Dexter et al., 2000) (164/287)	ns		
		(Kosuge et al., 2000) (44/81)	ns		
		(Kent et al., 2005) (72/220)	ns		
		(Polam et al., 2005) (102/177)	ns		
		(Mu et al., 2007) (54/95)	ns		
		(Redline et al., 2007) (69/129)		ns	
		(Andrews et al., 2008) (?/261)		ns	
		(Suppiej et al., 2009) (41/104)			*p* < 0.05 33 vs. 9% for speech delay
		(Hendson et al., 2011) (303/628)			coefficient = 3.93 (7.52–0.33) for MDI at 18 moths corrected age
		(Rovira et al., 2011) (87/177)			*p* = 0.03 18 vs. 5% for association of funisitis with severe disability
		(Helderman et al., 2012) (?/921)	ns		
		(Soraisham et al., 2013) (197/384)		ns	
		(Salas et al., 2013) (148/347)			RR 2.57 (1.02–6.46) for BSID III language score <70, RR 2.52 (1.11–5.72) for NDI
		(Nasef et al., 2013) (95/274)	ns		
		(Pappas et al., 2014) (910/2390)	ns		
		(Lee et al., 2014) (60/138)			β = −8.6 (−14.7 to −2.5) for language composite score
		(Ylijoki et al., 2016) (45/117)	ns	b = −7.22 (−14.31 to 0.13)	b = −1.29 (−2.40 to 0.18) for memory and learning functions in NEPSY
		(Bierstone et al., 2018) (145/350)	ns		
	Other premature infants *n* = 2	(Mittendorf et al., 2003) (21/121)	1.3 (1.1–1.9)		
		(Miyazaki et al., 2016) (1,235/4,078)		ns	
Clinical chorioamnionitis *n* = 15	VLBW/VLGA infants *n* = 8	(Wilson-Costello et al., 1998) (30/144)	3.79 (1.3-10.8)		
		(Dexter et al., 1999) (71/330)	ns		
		(Ambalavanan et al., 2000) (57/218)	ns		
		(Andrews et al., 2008) (?/261)	ns		
		(Rovira et al., 2011) (56/177)			*p* = 0.045 49 vs. 31% for association of chorioamnionitis with any disability
		(Nasef et al., 2013) (33/274)	0.2 (0.06–0.9)		0.3 (0.09–1.0) for language score below average, 0.2 (0.04–0.8) for motor score below average (Bayley-III)
		(Källén et al., 2015) (155/1,011)		ns	ns
		(Ylijoki et al., 2016) (16/117)	ns	ns	ns
	Other premature infants *n* = 7	(Hardt et al., 1985) (42/127)	*p* = 0.017 12 vs. 10%		
		(Morales, 1987) (92/698)	ns		
		(Vermeulen et al., 2001) (70/185)	ns		
		(Dammann et al., 2003) (36/294)		ns	
		(Versland et al., 2006) (13/130)		*p* = 0.04 difference of mean 11.0%	
		(Manuck et al., 2014) (384/1,771)	ns		
		(Vander Haar and Gyamfi-Bannerman, 2016) (194/157)	ns		ns
Clinical and/or histological chorioamnionitis *n* = 4	VLBW/VLGA infants *n* = 4	(Gray et al., 1997) (16/189)	ns		ns
		(Fung et al., 2003) (105/388)	ns		
		(Schlapbach et al., 2010) (33/99)	ns		ns
		(Pappas et al., 2014) (910/2390)	2.4 (1.3–4.3)		
	Other premature infants *n* = 0				

*Articles which analyzed histological and clinical chorioamnionitis separately are presented twice in the table. Articles including only very low birth weight (VLBW) infants (birth weight <1,501 g) and/or very low gestational age (VLGA) infants (born <32 weeks of gestation) are shown separately. The number of study subjects with chorioamnionitis in relation to the total number of study subjects are shown in parenthesis, “?” is used when the number of study subjects with abnormal blood flow patterns is not reported in the article.*.

Only seven of the 20 studies about histological chorioamnionitis demonstrated that histological chorioamnionitis was a risk factor for suboptimal development. Two studies found an association between histological chorioamnionitis and speech delay (Suppiej et al., 2009) and lower mental developmental index (Hendson et al., 2011) at 18 months of corrected age in very low birth weight and very low gestational age infants, but multivariate analyses were not performed in one of them (Suppiej et al., 2009). Rovira et al. (2011) found an association with severe disability in very low birth weight infants at 2 years of age in logistic regression analyses but gestational age was not included in the analyses. Two studies (Salas et al., 2013; Lee et al., 2014) found that histological chorioamnionitis and funisitis were associated with weaker language performance at 18–24 months of corrected age, but one of them (Salas et al., 2013) did not include gestational age in multivariate analyses. Mittendorf et al. (2003) found that funisitis predicted impaired neurodevelopment at 18 months of corrected age. Ylijoki et al. (2016) found that histological chorioamnionitis, but not funisitis associated with slightly weaker memory and learning functions as well as weaker cognitive performance at 5 years of age. Nine articles of histological chorioamnionitis (all of them discussed above) separately evaluated the effects of funisitis on the neurodevelopment of preterm born children. Four of these found that funisitis independently increased the risk for the developmental problems (Mittendorf et al., 2003; Redline et al., 2007; Rovira et al., 2011; Salas et al., 2013), while five studies did not find any associations (Redline et al., 2000; Helderman et al., 2012; Soraisham et al., 2013; Lee et al., 2014; Ylijoki et al., 2016).

Only five of 15 studies found an association between clinical chorioamnionitis and neurodevelopmental impairments in preterm born children. Hardt et al. (1985) found lower cognitive scores in children born after preterm rupture of membranes with chorioamnionitis compared to those without chorioamnionitis at 12 months corrected age. Wilson-Costello et al. (1998), Rovira et al. (2011), and Nasef et al. (2013) have reported an association between clinical chorioamnionitis and cognitive, verbal, and motor performance, as well as with neurological disability at 18–24 months of corrected age. Versland et al. (2006) had similar findings about increased risk for cognitive impairment up to 11 years of age.

In addition, there were four studies in which clinical and histological chorioamnionitis was not separated (Gray et al., 1997; Fung et al., 2003; Schlapbach et al., 2010; Pappas et al., 2014). One of these studies found an association between chorioamnionitis and lower cognitive scores at 18–22 months of corrected age (Pappas et al., 2014), while the others did not.

### Placental and Fetal Blood Flow

Eleven studies reported outcomes in association with umbilical artery blood flow. Seven of these did not find an association between abnormal flow in the umbilical artery and neurocognitive outcome (Valcamonico et al., 2004, 2007; Kirsten et al., 2007; Leppänen et al., 2009; Shand et al., 2009; Torrance et al., 2010; Eger et al., 2013). Male fetuses with intrauterine growth restriction and absent or reversed end diastolic flow in the umbilical artery have performed worse on cognitive tests than those with appropriate growth for gestational age (Morsing et al., 2011). Similarly, preterm neonates with absent or reversed end diastolic flow have been shown to have more cognitive, mental, and motor disabilities than appropriately grown controls (Vossbeck et al., 2001). Unfortunately, in both of these studies, blood flow patterns were not assessed in the groups of appropriately grown fetuses, which is a serious methodological limitation. Pathological flow in the umbilical artery has also been associated with moderate or severe neurological impairment in children with intrauterine growth restriction, but not in those with normal prenatal growth (Spinillo et al., 2005). Growth restricted preterm children with suboptimal neurological outcomes at 1 year of age had higher pulsatility index in the umbilical artery flow than those with normal neurodevelopmental outcome in univariate analyses (Kaukola et al., 2005).

An increased placenta-cerebral ratio (UA/MCA-ratio) reflecting brain sparing in a fetus has been associated with adverse cognitive performance in very low birthweight children (Leppänen et al., 2009). An increased UA/MCA ratio was associated with impaired cognitive outcomes at 5 years of age, but not with neurodevelopmental outcomes at 3 years of age in the same patient population (Scherjon et al., 1998, 2000).

Some studies have suggested that retrograde (Fouron et al., 2001, 2005) or abnormal blood flow in the aortic isthmus (Leppänen et al., 2009) is associated with non-optimal neurodevelopment. However, retrograde flow did not associate with abnormal outcomes in one study with six patients with retrograde flow (Kaukola et al., 2005). The results of the articles about placental and fetal blood flow are summarised in Table 2.

**Table 2:** The outcomes of the 15 included articles divided according to the placental or fetal blood flow measurements used (umbilical artery blood flow, increased placenta-cerebral ratio, retrograde flow in the aortic isthmus) and developmental outcomes (abnormal cognitive outcome ≤/>2 years of age or other).

			**Abnormal cognitive outcome ≤2 years of age**	**Abnormal cognitive outcome >2 years of age**	**Other**
			**OR (95% CI)**	**OR (95% CI)**	**OR (95% CI)**
Umbilical artery blood flow *n* = 11	VLBW/VLGA infants *n* = 7	(Eger et al., 2013) (38/71)		ns	
		(Morsing et al., 2011) (34/68)		*p* = 0.007 for FSIQ and *p* = 0.005 for VIQ in boys	
		(Valcamonico et al., 2007) (34/58)			ns
		(Kaukola et al., 2005) (?/17)			*p* = 0.005 for neurodevelopmental impairment at 1 year of corrected age
		(Shand et al., 2009) (39/119)	ns		
		(Vossbeck et al., 2001) (40/80)			*p* = 0.005 for cognitive development at 1–8 years of age
		(Leppänen et al., 2009) (17/83)	ns		
	Other premature infants *n* = 4	(Kirsten et al., 2007) (50/190)	ns	ns	*p* = 0.03 for performance subscale at 2 years of age
		(Valcamonico et al., 2004) (14/25)		ns	
		(Torrance et al., 2010) (?/71)	ns		
		(Spinillo et al., 2005) (75/266)			*p* = 0.05 for neurodevelopmental impairment at 12–24 months of age in growth restricted fetuses
Increased placenta-cerebral ratio *n* = 3	VLBW/VLGA infants *n* = 1	(Leppänen et al., 2009) (16/83)	*p* = 0.01		
	Other premature infants *n* = 2	(Scherjon et al., 1998) (34/96)			ns
		(Scherjon et al., 2000) (28/73)		*p* < 0.02, 54 vs. 20%	
Aortic isthmus blood flow *n* = 3	VLBW/VLGA infants *n* = 1	(Kaukola et al., 2005) (6/17)			ns
		(Leppänen et al., 2009) (?/83)	*p* = 0.03		
	Other premature infants *n* = 2	(Fouron et al., 2001) (5/44)		Relative risk 2.05 (1.49–2.83)	
		(Fouron et al., 2005) (?/48)			*p* = 0.007 for non-optimal neurodevelopmental outcome at 2–5 years of age

*Articles including only very low birth weight (VLBW) infants (birth weight <1,501 g) and/or very low gestational age (VLGA) infants (born <32 weeks of gestation) are shown separately. The number of study subjects with abnormal blood flow patterns in relation to the total number of study subjects are shown in parenthesis, “?” is used when the number of study subjects with abnormal blood flow patterns is not reported in the article.*.

### Maternal Smoking During Pregnancy

The four articles assessing the association between prenatal smoking exposure and cognitive and neurodevelopmental outcomes in preterm infants were based on the same cohort studied in Austria at 12 months of corrected age (Kiechl-Kohlendorfer et al., 2009), 24 months of corrected age (Kiechl-Kohlendorfer et al., 2010), and 5 years of corrected age (Kiechl-Kohlendorfer et al., 2013; Gnigler et al., 2015). Smoking information was based on maternal self-report after birth. The mothers who refused to report their smoking status were classified as smokers. These articles showed associations between prenatal smoking exposure and adverse developmental outcomes at 12 and 24 months of corrected age (Kiechl-Kohlendorfer et al., 2009, 2010), as well as an association with numerical skills and processing speed at 5 years of corrected age (Kiechl-Kohlendorfer et al., 2013; Gnigler et al., 2015). The results of these articles are summarized in Table 3.

**Table 3:** The outcomes of the four included articles about maternal smoking during pregnancy divided according the developmental outcomes (abnormal cognitive outcome ≤/>2 years of age or other).

	**Abnormal neurodevelopmental outcome ≤2 years of age**	**Abnormal cognitive outcome >2 years of age**
	**OR (95% CI)**	**OR (95% CI)**
(Kiechl-Kohlendorfer et al., 2009) (41/205)	*P* = 0.016	
(Kiechl-Kohlendorfer et al., 2010) (30/142)	3.36 (1.38–8.17)	
(Kiechl-Kohlendorfer et al., 2013) (34/135)		Delayed numerical skills: OR 4.26 (1.56–11.65)
(Gnigler et al., 2015) (44/161)		Reduced processing speed; OR 3.05 (1.43–6.52)

*All articles include only very low gestational age (VLGA) infants (born <32 weeks of gestation). The number of study subjects with prenatal smoking exposure in relation to the total number of study subjects are shown in parenthesis*.

## Discussion

There is conflicting data about the impact of prenatal risk factors on long-term development of preterm infants. It is challenging to do longitudinal studies extending from fetal risk factors or well-being to eventual long-term developmental outcomes, as so many of the studies have only small number of patients. Therefore, a summarizing, critical review of all the data is valuable.

A review of the effects of chorioamnionitis on the development of preterm infants helps clinicians get an overview of the heterogeneous studies with inconsistent results regarding this topic. Inconsistencies are partly due to small sample sizes, differences in patient populations, evaluation methods, and age points. This review classifies the studies according to the distinction between histological and clinical chorioamnionitis. Altogether, it seems that the majority of the publications do not support the belief that chorioamnionitis poses an independent risk for adverse development in preterm born children. This review does not give further support either to the hypothesis that clinical chorioamnionitis and funisitis are more deleterious to the developing central nervous system than histological chorioamnionitis.

One factor possibly explaining this complexity might be the maturation enhancing effects of chorioamnionitis on immature infants. Animal models have shown that chorioamnionitis significantly enhances lung maturation (Kramer et al., 2009), which might also be seen clinically in the lungs of preterm infants, although this comes with inflammatory consequences (Jobe, 2012). Therefore, the clinical effects of chorioamnionitis on the developmental outcome of preterm infants is complex. There are a few studies that show that histological chorioamniniotis has a protective effect on mortality rates (Hendson et al., 2011) and neurodevelopment of preterm infants when compared with placental underperfusion (van Vliet et al., 2012). Therefore, it seems that chorioamnionitis might have beneficial effects, in addition to the deleterious effects, on the developing preterm infant. Another factor modifying the effects of chorioamnionitis might be prenatal glucocorticoids. Most preterm infants are exposed to the anti-inflammatory effects of prenatal glucocorticoids. It is likely that this immunomodulation attenuates the effects of chorioamnionitis. Indeed, a meta-analysis has shown that prenatal steroid administration is associated with a reduced risk for brain lesions in clinical and histological chorioamnionitis (Been et al., 2011). One study found that histological chorioamnionitis was associated with CP only in those infants who had not been given two doses of prenatal corticosteroids (Kent et al., 2005). However, our review also includes recent publications with patients who had a high prenatal glucocorticoid administration rate where chorioamnionitis still seemed to be a significant risk factor for suboptimal development.

We can conclude that the available evidence does not suggest chorioamnionitis is a major independent risk factor for suboptimal cognitive and neuropsychological development in preterm born children. As there are no “healthy” preterm controls without other risk factors, we can only conclude that chorioamnionitis may not be a greater risk for the brain of a preterm infant than other underlying pathologies already present before preterm delivery.

Most of the data on the association of prenatal fetoplacental blood flow and later neurocognitive development is focused on umbilical artery blood flow. The pulsatility index of umbilical artery flow reflects the number of tertiary villous arterioles in the placenta (Acharya et al., 2004). Thus, pulsatility index of the umbilical artery flow is known to increase in placental insufficiency. Most of the studies did not find an association between increased pulsatility in the umbilical artery flow and later neurocognitive outcomes in preterm born infants. However, there are some data on abnormal umbilical blood flow and impaired neurodevelopment in fetuses suffering from placental insufficiency (Kaukola et al., 2005). However, the sample sizes were often small and methodological problems complicated the interpretation of the data.

The so called “brain sparing” effect has been considered protective to fetuses with growth restriction. Thus, it is interesting that an increased placenta-cerebral ratio has been associated with impaired cognitive outcomes in some (Scherjon et al., 2000; Leppänen et al., 2009), although not all studies (Scherjon et al., 1998). The data is too sparse to draw conclusions on the effect of aortic isthmic blood flow on infant neurocognitive development.

Prenatal smoking exposure has been associated with many adverse effects on fetal health as well as an increased risk for diseases in later life. The data show a negative association between prenatal smoking exposure and later cognition in preterm infants up to 5 years of age. However, it is challenging to prove a causal link between prenatal smoking exposure and cognitive outcomes in later life because genetic and familial factors are known confounders (Gilman et al., 2008; Knopik, 2009). Maternal education, which is one important familial factor, was adjusted for in the analyses of these studies with preterm infants (Kiechl-Kohlendorfer et al., 2009, 2010, 2013; Gnigler et al., 2015). The familial factors include also disadvantageous parenting and family functioning. Therefore, more precise measurements of genetic and familial influences should be taken into account (e.g., by sibling design). It has been shown in a sibling design study that the unexposed sibling was also at an increased risk of poor school performance at the age of 15 years Lambe et al. (2006). On the other hand, there is a large population study showing that the effects of prenatal smoking exposure remained also in a sibling design study (Ekblad et al., 2017). Individual genetic polymorphisms might also modify the effect of prenatal smoking exposure on cognitive functioning (Morales et al., 2009). Women who smoke during pregnancy may also be more likely to engage in other unhealthy behaviors, like alcohol drinking during pregnancy when smoking serves as an indicator for unhealthy lifestyle. A Finnish study showed that heavy smoking before pregnancy was associated with lower cognitive scores in children aged 56 months even if the mother did not smoke during pregnancy (Heinonen et al., 2011). The authors of the aforementioned study speculate that the association might be explained by maternal smoking-related health habits and status. In future studies, it would be interesting to also control also for cognitive abilities and executive functions of the parents.

The lack of reliable validation of smoking exposure is one significant limitation in most of the studies on the effects of smoking exposure. Smoking exposure can be verified by measuring cotinine, a metabolite of nicotine, in the saliva or hair of a mother during pregnancy (Shipton et al., 2009). Cotinine measurements would also reveal a significant environmental smoking exposure. In addition, there might be different effects of smoking exposure at different stages of pregnancy, and so evaluating changes in cotinine levels throughout pregnancy might be relevant.

One limitation of this review is the heterogeneous and rather small patient populations in the original articles. The inclusion criteria vary between the studies, and thus the comparison of the studies is difficult. The same applies to the outcome variables and time points. Also, some of the studies were done in the 1980s and 1990s, and it is well known that the treatments and outcomes of preterm infants have developed during the subsequent years.

This review summarizes the data on several prematurity related prenatal risk factors which play a role in the developmental outcomes of preterm infants. To optimize the developmental outcomes of this patient population we need to first optimize the fetal well-being before birth. More longitudinal research with large patient populations that extends from the fetal life to long-term developmental outcomes is needed. To draw definite conclusions about clinical practices such as the right timing of the delivery requires randomized controlled trials. It is also crucial to implement all practices which protect brain development and improve later neurodevelopmental outcomes of immature preterm infants.

## Author Contributions

MY, EE, ME, and LL took part in the design of the study, the interpretation of data, and drafted the initial manuscript. All authors have approved the final manuscript as submitted and agree to be accountable for all aspects of the work.

### Conflict of Interest Statement

The authors declare that the research was conducted in the absence of any commercial or financial relationships that could be construed as a potential conflict of interest.
